# Bio-Based Solutions for Agriculture: Foliar Application of Wood Distillate Alone and in Combination with Other Plant-Derived Corroborants Results in Different Effects on Lettuce (*Lactuca Sativa* L.)

**DOI:** 10.3390/biology11030404

**Published:** 2022-03-05

**Authors:** Riccardo Fedeli, Andrea Vannini, Massimo Guarnieri, Fabrizio Monaci, Stefano Loppi

**Affiliations:** 1 Department of Life Sciences, University of Siena, 53100 Siena, Italy; riccardo.fedeli@student.unisi.it (R.F.); andrea.vannini@unisi.it (A.V.); massimo.guarnieri@unisi.it (M.G.); fabrizio.monaci@unisi.it (F.M.); 2 BAT Center—Interuniversity Center for Studies on Bioinspired Agro-Environmental Technology, University of Naples ‘Federico II’, 80138 Napoli, Italy

**Keywords:** biomass, chlorophyll, starch, sugars, wood vinegar

## Abstract

**Simple Summary:**

Bio-stimulants are showing growing success and are gradually replacing synthetic fertilizers in agriculture. Wood distillate (WD), also known as wood vinegar or pyroligneous acid, is a by-product of biomass pyrolysis and is increasingly used as a bio-stimulant for crop production. The purpose of this work was to test two types of WD utilizations: (i) pure WD (from BioDea^®^) and (ii) WD combined with 3% soy lecithin and 5% flavonoid-rich wood glycolic extract (BF; BioDea Flavor^®^), at concentrations of 0.25% and 0.50%. Our results indicate that foliar applications of both WD and BF increased chlorophyll content, biomass, and starch content in the treated lettuce, while WD, at a concentration of 0.50%, also increased glucose and fructose content. All the treatments were found to be safe, as neither of them showed a significant increase in the content of potentially toxic elements (PTEs) in lettuce.

**Abstract:**

Bio-stimulants are showing growing success and are gradually replacing synthetic fertilizers in agriculture. Wood distillate (WD), also known as wood vinegar or pyroligneous acid, is a by-product of biomass pyrolysis and is increasingly used as a bio-stimulant for crop production. This study investigated whether weekly foliar applications of 0.25% and 0.50% WD have a differential effect on the chlorophyll and sugar content as well as biomass production in lettuce (*Lactuca sativa* L.). Moreover, the additional beneficial effect from the addition of corroborants of plant origin such as 3% soy lecithin and 5% flavonoid-rich wood glycolic extract to WD (BF) was investigated. Moreover, the possible toxicological concern from some potentially toxic elements (PTEs), namely Cd, Cu, Fe, Pb, and Zn, which may be abundant in WD was verified. After four weeks, we found that 0.25% WD not only increases lettuce biomass, which has an economic value, but also has beneficial effects on other qualitative parameters such as sugars and total sweetness. On the other hand, the use of 0.5% WD decreased the content of soluble sugars, suggesting a hormetic-type effect. We did not find evidence of further beneficial effects from the addition to WD of plant-derived corroborants, nor of any enrichment in the content of the investigated PTEs.

## 1. Introduction

Chemical fertilizers have played an important role in boosting crop productivity and enabling a growing population to be fed without using additional land [1]. However, their long-term and excessive use has become a major environmental concern [2], and at present there is a growing interest in bio-based alternatives to chemical fertilizers [3].

Wood distillate (WD), also known as pyroligneous acid, is a by-product of biomass pyrolysis for energy production [4,5] and is known to be very rich in different molecules, such as esters, alcohols, acids, sugars, and phenols [6,7]. There is convincing evidence that WD has great potential in agriculture [4,8,9] because of its ability to act as a bio-stimulant for crops, to increase biomass [10,11] and fruit production (quality, size, and weight) [6,12]. Moreover, ecotoxicological studies have confirmed that this product is safe for the environment since it has been shown to have no adverse effects on either aquatic or soil organisms [13,14], as well as non-target bioindicators, i.e.**,** lichens, mosses, and aquatic ferns [15,16]. Additionally, an investigation of the safety profile of wood distillate excluded inflammatory and cytotoxic effects at low recommended doses and short-time applications at higher doses [17].

Recently, WD has been included in the list of products that can be used in Italy in organic farming, as well as in combination with other plant-derived corroborants such as soy lecithin and a flavonoid-rich wood glycolic extract, i.e.**,** a product derived from the extraction of wood with water and glycerin [18].

Lettuce (*Lactuca sativa* L.) has been used as a model crop species to test the effect of 0.2% WD, with and without the addition of 3% soy lecithin, on the ability to increase the photosynthetic performance and the growth of this horticultural plant [19]. The results of this study showed that WD has a positive effect on chlorophyll content (+54%) and biomass production (+39%) and that the addition of lecithin further increased biomass production (+51%).

Based on the indications of the producers, i.e., that WD can be applied at concentrations in the range of 0.2–0.5%, the first aim of this paper was to test whether different doses of WD have a differential effect on the chlorophyll and sugar content as well as the biomass production in lettuce. Moreover, a second aim was to test whether the addition of 3% soy lecithin and 5% flavonoid-rich wood glycolic extract to WD has a further positive effect on the above parameters. Additionally, the potential toxicological concern regarding treatments with WD was investigated by checking the concentration of some potentially toxic elements (PTEs), namely Cd, Cu, Fe, Pb, and Zn, which may be abundant in WD [16]. Optimizing the use of WD according to crop needs may provide farmers with the most efficient use of this bio-stimulant and may increase the economic return of agricultural production.

## 2. Materials and Methods

### 2.1. Experimental

Seedlings of *Lactuca sativa* (cv. ‘Adela’), bought from a local nursery, were sown and grown together in polystyrene phytocells in a greenhouse and characterized by an average height of 15 cm. In the laboratory, the plants were transplanted into plastic pots (10 × 10 × 12 cm) using potting soil prepared in the Botanical Garden of the University of Siena (main soil characteristics are provided in Table 1) and then left to acclimatize for one week in a climatic chamber at temperature = 20 ± 1 °C, relative humidity (RH) = 60 ± 2%, light = 400 µmol m**^−^**^2^ s**^−^**^1^ PAR, and photoperiod = 12 h. Seedlings were treated four times per week with foliar applications (spray) of either mineral water (control), sweet chestnut (*Castanea sativa*) WD (BioDea**^®^**, Arezzo, Italy), or WD with the addition of 3% soy lecithin and 5% flavonoid-rich wood glycolic extract (hereafter BF; BioDea Flavor**^®^**, Arezzo, Italy) at concentrations of 0.25% and 0.50%. Analysis of the WD and the BF provided by the producer indicated that the pH was in the range of 3.5–4.5, density was 1.05 kg/L, acetic acid was in the range of 2–2.3%, and polyphenols were in the range of 22–25 g/L.

The treatment solutions (100 mL for each set of six seedlings, statistical replicates) were sprayed over the whole plant in the late afternoon, following the procedure described by Vannini et al. [19]. After the treatment, the seedlings were left in the climatic chamber at the same conditions as described above, randomly rotating their position every two days to minimize possible micro-environmental effects. The experiment lasted 4 weeks and was replicated 3 times.

### 2.2. Chlorophyll Content

Measurements of the total chlorophyll content (10 per plant) were carried out using a chlorophyll content meter (CCM-300, Opti-Science, Hudson, IN, USA), which allowed for estimations of the chlorophyll content without damaging the plant leaves. For each plant, measurements were taken at the apical parts of the three major leaves, avoiding leaf nerves. The results are expressed on a surface basis [20].

### 2.3. Starch Content and Soluble Sugars

Lettuce leaves were removed, dried in a ventilated oven at 30 °C for two days, and pulverized with mortar and pestle. The starch content was determined following the method described by Loppi et al. [21]. Ground samples (50 mg) were homogenized in 2 mL of dimethyl sulfoxide (DMSO). Then 0.5 mL of 8 M HCl was added and samples were placed in a ventilated oven for 30 min at 60 °C. After cooling, 0.5 mL of 8 M NaOH and 7 mL of deionized water were added. Samples were then centrifuged at 4000 rpm for 5 min, and 0.5 mL of supernatant was added to 2.5 mL of Lugol’s solution (HCl 0.05 M, 0.03% I_2_, and 0.06% KI). After 15 min, samples were read at 605 nm with a UV-VIS spectrophotometer (Agilent 8453). Quantification was run using a calibration curve (10–400 μg/mL) prepared with pure starch (Merck). The results are expressed on a fresh-weight basis (mg/g FW).

For the determination of the content of soluble sugars, ground samples (100 mg) were homogenized in 2 mL of deionized water and then centrifuged at 15,000 rpm for 5 min. The supernatant was filtered at 0.45 μm using a syringe filter and then directly analyzed using an HPLC (Waters 600 system, MA, USA) equipped with a Waters 2410 refractive index detector. Sugar separation was allowed using deionized water as mobile phase, eluted at 0.5 mL/min, and a Waters Sugar-Pak I ion-exchange column (6.5 × 300 mm) kept at 90 °C using an external temperature controller (Waters Column Heater Module, MA, USA). Sugar quantification was obtained using calibration curves prepared by dissolving analytical sugars (Sigma) in deionized water at concentrations of 0.1–20 mg/mL. The precision of the analysis, estimated by the coefficient of variation of 5 replicates, was always >95%. Recoveries were in the range of 94–105%. Results are expressed on a fresh weight basis (mg/g FW).

The total sweetness index (TSI) was calculated according to the formula proposed by Clarke [22]:

*TSI* = (1.00 × [*sucrose*]) + (0.76 × [*glucose*]) + (1.50 × [*fructose*])

### 2.4. Dry Biomass

Lettuce leaves were removed from the plant and oven-dried at 105 °C for 3 h. Afterwards, samples were left to stabilize for five minutes and then weighed with a precision balance.

### 2.5. Chemical Analysis

Ground samples (200 mg) were dissolved in 3 mL of 70% HNO_3_ and 0.5 mL of 30% H_2_O_2_ using a microwave-digestion system (Milestone Ethos 900, Bergamo, Italy) at 280 °C and 55 bar [23]. The content of Cd, Cu, Fe, Pb, and Zn was quantified by ICP-MS (Perkin Elmer NexION 350, MA, USA). Analytical quality was verified using the certified reference material NCS DC 73,350 ‘Poplar leaves’, which indicated recoveries in the range of 92–112%. Precision of the analysis was estimated by the coefficient of variation of 5 replicates and was always >98%. Results are expressed on a fresh weight basis (μg/g fw).

### 2.6. Statistical Analysis

Since not all variables matched a normal distribution, a non-parametric approach was adopted [24], with the parameter estimates being expressed by their median value and the associated error as the interquartile range divided by the square root of the number of observations. The significance of differences (*p* < 0.05) between the control and the treated samples was verified with a pairwise permutation t-test, correcting for multiple testing according to Benjamini and Hochberg [25]. All calculations were run using the R software [26].

## 3. Results

**Figure 1:**
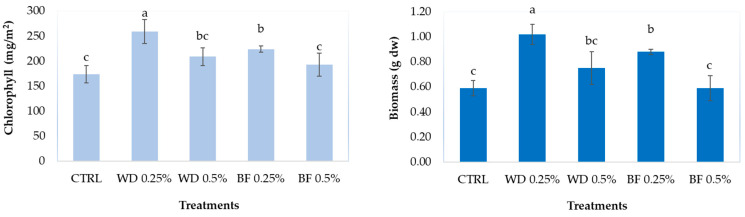
Chlorophyll content and biomass (median ± error) of lettuce leaves after four weekly foliar treatments with water (CTRL), 0.25% and 0.5% WD (BioDea Wood Distillate), and BF (BioDea Flavor). Different letters indicate statistically significant (*p* < 0.05) differences between treatments.

Weekly foliar applications of 0.25% WD and BF increased both the content of chlorophyll and the amount of biomass produced (mean water content was 96%), with WD showing the highest efficiency; no statistically significant effect was found for the treatments at 0.50% concentrations (Figure 1).

The starch content of lettuce leaves was increased by both treatments with 0.25% and 0.50% WD and BF (Table 2). On the contrary, these treatments did not show any effect on the content of sucrose and pectin (Table 2). The response of glucose, fructose, and TSI was more complex, with a decrease compared to the control values after treatment with 0.50% WD, a remarkable increase (14-fold for the sugars, 8-fold for TSI) with 0.25% WD, and no effect at all for both treatments with BF (Table 2).

The application of WD and BF did not alter the content of Cd, Cu, Fe, Pb, or Zn in lettuce leaves (Table 3).

## 4. Discussion

Among the tested treatments, the foliar application of 0.25% WD produced the most positive effects on lettuce. Specifically, this treatment increased the content of chlorophyll, starch, soluble sugars, and biomass, consistently with the results obtained by Vannini et al. [19], which showed increases in the chlorophyll and dry biomass using 0.2% WD.

Chlorophyll is a fundamental molecule for plants, responsible for the functionality of photosynthesis and thus related to energy production and plant growth, which is why an increase in chlorophyll also leads to an increase in the produced biomass [27]. Our results with 0.25% WD and BF showed a chlorophyll increase by 29–49%, and similar results have also been reported for mustard and rice after foliar applications of 0.2% WD [10,28], as well as in 4-week-old rice seedlings treated with 0.33% WD [11]. The 49–73% biomass increase observed in our lettuce plants treated with 0.25% WD and BF is consistent with similar increases reported for lettuce (+42%, [7]), tomato (27%, [12]), and rice (+20–45%, [10]) following the foliar application of 0.13–0.2% WD. Interestingly, no effect was found for the 0.5% treatments with either WD or BF, suggesting a hormetic-type effect of these bio-stimulants.

Some studies reported an increase in soluble sugar in crop plants after the application of WD, e.g., sweet pepper [29], tomato [30], and eggplant [31]. Using soluble sugars as an indicator of plant productivity, we argue that an increase in their content is likely linked to increased photosynthetic performance and consequent plant yields. As a matter of fact, it is well-known that sugars are fundamental to the stimulation of cell wall synthesis and the interaction with auxins [32,33]. Our results show a notable ca. 1400–1500% increase in glucose and fructose, as well as a consequent 770% increase in TSI after treatment with 0.25% WD, while no effect was found for sucrose. Moreover, BF did not cause any effect, both at 0.25% and 0.5%, while the application of 0.5% WD reduced the content of glucose, fructose, and TSI by ca. 30%. It is possible that this latter dilution is not suitable for lettuce but more suitable for more resistant horticultural crops, i.e., plants with a greater leaf thickness or a thicker waxy cuticle layer, such as cabbage and cauliflower [34]. In the literature, there are reports of no effect of WD treatment [35] or even a decrease in leaf-soluble sugar content [36]. Nevertheless, besides acting as a bio-stimulant, WD is also known to counteract plant pathogens, such as fungi and bacteria [37,38], and antibacterial activity of wood distillate has been found at concentrations as low as 0.4% [39]. Notwithstanding the fact that low doses (high dilutions) of wood distillate can stimulate plant growth and development [6,7], there is also evidence that much higher doses (lower dilutions) can cause plant senescence, thus suggesting its potential use as an herbicide [14]. Our results for soluble sugars further suggest a hormetic-type effect of WD, and the mechanism behind this response deserves further investigation.

All treatments showed a significant > 100% increase in the leaf content of starch, indicating that all of them succeeded in increasing the energy storage for the cellular metabolism. Starch is the main energy reserve of plants, composed of the two glucose polymers amylose and amylopectin [40], which can be accumulated and released in the form of glucose and maltose during the growth phases of the plant [41] or locally for specific processes such as nectar production [42]. Studies on the effect of WD on starch are scanty: Sun-Ok and Dong-Hoon [43] found that WD concentrations of up to 0.5% decreased the leaf starch content of the orchid *Neofinetia falcata* but increased its root content. We have not assayed the root parts of the plant, but the starch content of our control lettuce leaves was consistent with that reported in other studies [44].

From a toxicological point of view, all treatments did not alter the content of the investigated elements in lettuce leaves, confirming the results obtained by Fačkovcová et al. [15,16] about the environmental safety of WD. The concentrations of Fe, Zn, and Cu of our samples were within the common ranges reported in the literature: 1.97–8.6 mg/kg for Fe [45,46], 2.1–9.4 mg/kg for Zn [45,46,47], and 0.23–1.4 mg/kg for Cu [45,47]. Moreover, the concentration of Cd and Pb measured in the treated lettuce plants were well below (one order of magnitude below) the threshold limit established by the European Union for the marketability of broadleaf horticultural crops, i.e., 0.1 and 0.2 mg/kg fw [48], respectively.

## 5. Conclusions

The use of bio-based products such as bio-stimulants is presently being widely investigated in the search for solutions to agricultural problems. Here, we have shown that weekly foliar applications of 0.25% WD not only increase lettuce biomass, which has economic value, but have beneficial effects also on other qualitative parameters such as sugars and total sweetness. On the other hand, the use of 0.5% WD decreased the content of soluble sugars, suggesting a hormetic-type effect of this bio-stimulant. We did not find evidence of further beneficial effects from the addition to WD of plant-derived corroborants such as soy lecithin and flavonoid-rich wood glycolic extract. Additionally, we did not find evidence of any enrichment in the content of some PTEs, namely Cd, Cu, Fe, Pb, and Zn, which may be abundant in WD.

Since WDs produced from different types of wood and under different physical conditions can have different chemical characteristics, it remains to be investigated whether our results can be generalized to other WDs.

## Figures and Tables

**Table 1:** Soil characteristics (mean ± standard error).

Soil Characteristics	
pH	8.07 ± 0.01
CaCO_3_ (%)	20.3 ± 0.3
Carbon (%)	1.8 ± 0.1
Nitrogen (%)	1.6 ± 0.1
Ca^2+^ (mg/kg)	4013 ± 19
Mg^2+^ (mg/kg)	124 ± 1
Na^+^ (mg/kg)	324 ±16
K^+^ (mg/kg)	168 ± 4
CEC (meq/100 g)	23 ± 0.1

**Table 2:** Content of starch and soluble sugars (mg/g fw, median ± error), and total sweetness index (TSI) in lettuce leaves after four weekly foliar treatments with water (CTRL), 0.25% and 0.5% wood distillate (WD) and WD combined with 3% soy lecithin and 5% flavonoid-rich wood glycolic extract (BF). Different letters indicate statistically significant (*p* < 0.05) differences between treatments.

	Starch	Sucrose	Glucose	Fructose	Pectin	TSI
CTRL	7 ± 0.7 a	0.27 ± 0.05 a	0.25 ± 0.01 a	0.25 ± 0.01 a	8.5 ± 0.4 a	0.93 ± 0.02 a
WD 0.25%	15 ± 0.8 b	0.23 ± 0.03 a	3.49 ± 0.67 c	3.47 ± 0.62 c	9.0 ± 0.3 a	7.16 ± 1.67 c
WD 0.50%	15 ± 1.7 b	0.22 ± 0.04 a	0.18 ± 0.02 b	0.18 ± 0.01 b	10.2 ± 1.6 a	0.66 ± 0.06 b
BF 0.25%	16 ± 2.2 b	0.27± 0.04 a	0.26 ± 0.06 a	0.27 ± 0.05 a	11.0 ± 1.1 a	0.80 ± 0.08 a
BF 0.50%	16 ± 1.4 b	0.28 ± 0.03 a	0.23 ± 0.02 a	0.36 ± 0.06 a	11.0 ± 1.3 a	1.20 ± 0.13 a

**Table 3:** The content of potentially toxic elements in lettuce leaves (mg/kg fw, median ± error) after four weekly foliar treatments with water (CTRL), 0.25% and 0.5% wood distillate (WD), and WD added with 3% soy lecithin and 5% flavonoid-rich wood glycolic extract (BF). For all elements, no statistically significant differences between treatments were found.

	Cd	Cu	Fe	Pb	Zn
CTRL	0.007 ± 0.01	0.44 ± 0.08	3.5 ± 0.3	0.022 ± 0.002	4.4 ± 0.5
WD 0.25%	0.008 ± 0.01	0.50 ± 0.07	3.6 ± 0.4	0.022 ± 0.003	3.6 ± 0.2
WD 0.50%	0.008 ± 0.01	0.40 ± 0.04	4.3 ± 0.7	0.025 ± 0.009	3.4 ± 0.4
BF 0.25%	0.009 ± 0.01	0.52 ± 0.06	3.9 ± 0.4	0.021 ± 0.006	4.4 ± 0.5
BF 0.50%	0.008 ± 0.01	0.52 ± 0.05	3.5 ± 0.3	0.022 ± 0.007	4.0 ± 0.5

## Data Availability

The raw data presented in this study are available on request from the corresponding author.
